# Hematoimmunological responses of juvenile Nile tilapia (*Oreochromis niloticus*) receiving the dietary supplementation of immunomodulators and different levels of vitamins after challenge with physical stress

**DOI:** 10.29374/2527-2179.bjvm001124

**Published:** 2024-07-17

**Authors:** Domickson Silva Costa, Scheila Anelise Pereira Dutra, Iracema Lima Pereira, Lucas Cardoso, Paula Brando de Medeiros, Liseth Vanessa Perenguez Riofrio, Maria Clara Miguel Libanori, Thiago Augusto Soligo, Eduardo Yamashita, Ulisses de Pádua Pereira, José Luiz Pedreira Mourinõ, Maurício Laterça Martins

**Affiliations:** 1 Fisheries engineer, AQUOS-Aquatic Organisms Health Laboratory, Aquaculture Department, Universidade Federal de Santa Catarina (UFSC), Florianópolis, SC, Brazil.; 2 Aquaculture engineer, LCM-Marine Shrimp Laboratory, Aquaculture Department, UFSC, Florianópolis, SC, Brazil.; 3 Aquaculture engineer, AQUOS-Aquatic Organisms Health Laboratory, Aquaculture Department, UFSC, Florianópolis, SC, Brazil.; 4 Veterinarian, AQUOS-Aquatic Organisms Health Laboratory, Aquaculture Department, UFSC, Florianópolis, SC, Brazil.; 5 Aquaculture engineer, DSM-Nutritional Products Costa Rica, Heredia, Costa Rica.; 6 Zootechnist, DSM-Nutritional Products, Brazil S.A, São Paulo, Brazil.; 7 Veterinarian, LABBEP- Department of Preventive Veterinary Medicine, Laboratory of Bacteriology in Fish, UEL, Londrina, PR, Brazil.; 8 Zootechnist, AQUOS-Aquatic Organisms Health Laboratory, Aquaculture Department, UFSC, Florianópolis, SC, Brazil.; 9 Biologist, AQUOS-Aquatic Organisms Health Laboratory, Aquaculture Department, UFSC, Florianópolis, SC, Brazil.

**Keywords:** immune system, immunomodulatory effect, synergy, cichlid, sistema imunológico, efeito imunomodulador, sinergia, ciclídeo

## Abstract

In this study, we analyzed the hematoimmunological effects of dietary supplementation with immunomodulators (β-glucans + nucleotides) and different levels of vitamins on Nile tilapia (*Oreochromis niloticus*) after exposure to physical stress. The following four diet treatments were used: diets with indicated vitamin levels (Vitind), diets with Vitind + immunomodulator (Vitind + Immune), diets with high vitamin content (Vithigh), and those with Vithigh + immunomodulator (Vithigh + Immune). The experiment included 560 fish in 28 tanks (20 fish tank^-1^), with seven replicates per treatment. After 60 days of supplementation, the water temperature was set at 20 °C, and complete biometrics were performed. The animals were then subjected to physical stress with temperature oscillations of 20 ºC to 30 ºC/30 ºC to 20 ºC/20 ºC to 30 ºC. Hematoimmunological data from 140 animals were collected post-stress. Antimicrobial titer and total plasma protein levels were significantly higher in fish not receiving immunomodulator-supplemented diets (2.88 ± 0.43 log2 and 26.81 ± 4.01 mg∙mL^−1^, respectively) than in those that did. Conversely, the agglutination titer increased in fish fed with lower vitamin levels (3.33 ± 0.66 log2) compared to those with higher vitamin levels. Increased immunoglobulin levels were observed in fish fed diets co-supplemented with vitamins and immunomodulators, revealing an interaction between immunomodulators and dietary vitamin levels. In summary, the inclusion of immunomodulators in the diet enhanced the animals’ resistance to physical stress and improved hematoimmunological parameters. Additionally, a high vitamin content in the diet did not modulate the immune responses in the animals.

## Resumo

Neste estudo analisamos os efeitos hematoimunológicos da suplementação dietética com imunomoduladores (β-glucanos+nucleotídeos) e diferentes níveis de vitaminas na tilápia do Nilo (*Oreochromis niloticus*) após exposição ao estresse físico. Foram utilizados quatro tratamentos: dietas com níveis indicados de vitaminas (Vitind), dietas com Vitind + imunomodulador (Vitind+Immune), dietas com alto teor de vitaminas (Vithigh) e dietas com Vithigh + imunomodulador (Vithigh+Immune). O experimento incluiu 560 peixes em 28 tanques (20 peixes tanques^-1^), com sete repetições por tratamento. Após 60 dias de suplementação, a temperatura da água foi fixada em 20 °C e realizada biometria completa. Os animais foram submetidos a estresse físico com oscilações de temperatura de 20 ºC a 30 ºC/30 ºC a 20 ºC/20 ºC a 30 ºC. Dados hematoimunológicos de 140 animais foram coletados pós-estresse. O título antimicrobiano e os níveis de proteína plasmática total foram significativamente maiores em peixes que não receberam dietas com imunomodulador (2,88±0,43 log2 e 26,81±4,01 mg∙mL^−1^) do que naqueles que receberam. Por outro lado, o título de aglutinação aumentou em peixes alimentados com níveis mais baixos de vitaminas (3,33±0,66 log2) comparado àqueles com níveis mais elevados. Níveis aumentados de imunoglobulinas foram observados em peixes alimentados com dietas co-suplementadas com vitaminas e imunomoduladores, revelando interação entre imunomoduladores e níveis de vitaminas na dieta. Em resumo, a inclusão de imunomoduladores na dieta aumentou a resistência dos animais ao estresse físico e melhorou os parâmetros hematoimunológicos. Além disso, o alto teor de vitaminas na dieta não modulou as respostas imunológicas dos animais.

## Introduction

*Oreochromis niloticus* is the third most cultivated fish species worldwide (Prabu et al., 2019) and the most cultivated in Brazil. According to the Associação Brasileira da Piscicultura (2024), of the aquatic organisms cultivated in Brazil in 2023, tilapia contributed to 65.3% of the total produce. The cultivation of tilapia is attractive because of its high production capacity in intensive and super-intensive systems (Brito et al., 2017), hardiness during handling, and resistance to changes in temperature (El-Sayed & Kawanna, 2008), dissolved oxygen (DO), and salinity and to certain diseases. It is an omnivorous fish that readily accepts exogenous food, has a high growth rate, and is widely consumed (El-Sayed, 2005; Barroso et al., 2015; Siqueira et al., 2021).

Despite the rusticity and resistance of this species, its susceptibility to some parasitic, fungal, or bacterial infections affects its cultivation. The parasites infecting tilapia include *Ichthyophthirius multifiliis*, *Trichodina* sp., *Ambiphyra* sp., *Apiosoma* sp., monogeneans, and digeneans (Zanolo & Yamamura, 2006; Silva et al., 2021). Moreover, the major bacterial species infecting tilapia are *Edwardsiella tarda*, *Francisella noatunensis orientalis*, *Streptococcus agalactiae*, *Streptococcus iniae*, and *Aeromonas* spp. (Figueiredo & Leal, 2008). In addition, it is faced with inadequate management and low temperatures, particularly during winters in subtropical regions (Zerai et al., 2010; Nobrega et al., 2020). Generally, in these regions, between the end of autumn and the beginning of spring, the culture waters register low temperatures, between 11 and 13 ºC, values close to lethality for species such as Nile tilapia, as its thermal comfort range is between 26 and 30 ºC (Altun et al., 2006; Kubitza, 2006; Zadinelo et al., 2020).

Diseases, nutritional deficiency, the temperature falls and the oscillation of temperature in winter, stocking density, and neuroendocrine factors affect the immune system of fish, making them less tolerant to handling and thereby causing mortality (Kubitza, 2006; Falcon et al., 2007; Zadinelo et al., 2020). Antibiotics and chemotherapy have been used as prophylactic and treatment measures to prevent these losses (Ding & He, 2010; Wang et al., 2019). However, their indiscriminate use can lead to the accumulation of antibiotics and chemotherapy drugs in fish tissues and the emergence of “super-bacteria,” which do not respond to antibiotic treatments (Akanmu, 2018; Kraemer et al., 2019).

Owing to the problems associated with the indiscriminate use of antibiotics and the abovementioned unfavorable factors, it is necessary to develop new products to remedy their effects. Examples are vitamins, implicated in various physiological and metabolic processes, growth, health, and reproduction (McDowell, 2000; El‐Sayed & Izquierdo, 2021), and immunomodulators, which improve the immune system and neutralize the immunosuppressive state, increasing natural resistance (Bagni et al., 2005; Abdelrazek et al., 2017). Immunomodulators are natural or chemically synthesized compounds used to modulate and enhance the immune resistance of organisms (Ching et al., 2020; Gannam & Schrock). Their components and by-products include a variety of chemical agents, polysaccharides, mannan oligosaccharides, animal extracts, and plant extracts (Abdelrazek et al., 2017). Examples of these substances include nucleotides and β-glucans (Xu et al., 2015; Dawood et al., 2020).

β-glucan is a structural polysaccharide formed by a glucose block found in the cell walls of bacteria, fungi, yeasts, protozoa, and plants (Wismar et al., 2010; Selim & Reda, 2015). Its structure comprises β-(1,3) and β-(1,6) bonds in a non-repetitive and non-random order, with side chains of varying lengths (Chagas et al., 2013; Aramli et al., 2015). β-glucans stimulate the innate defense mechanism, increase the phagocytic activity of macrophages, fight pathogens and stress (Ringø et al., 2016; Lu et al., 2019; Penney et al., 2019), and stimulate biological activities such as antimicrobial, antioxidant, and anti-inflammatory effects (Dawood et al., 2020).

Briefly, β-glucans bind to and activate phagocytic cells to produce cytokines, promote a chain reaction, and increase the production of phagocytes, alerting the immune system to fight pathogens, stress, and environmental challenges, acting as a prophylactic agent that increases immune resistance. These peptides have great potential for use in aquaculture in enhancing resistance against pathogens and stress and as an alternative to antibiotics and chemotherapy (Pohlenz & Gatlin 3rd, 2014; Watts et al., 2017; Dawood et al., 2018; Yamamoto et al., 2018).

Nucleotides, like β-glucans, have both plant and animal origins and can, therefore, be found in yeast cells (Gil, 2002; Fegan, 2006). These are intracellular compounds with low molecular weight, a nitrogenous purine or pyrimidine base, a pentose sugar, and one or more phosphate groups (Gil, 2002). According to Li et al. (2015), Nelson & Cox (2018), and Bowyer et al. (2019), nucleotides are used as the structural components of enzymatic cofactors and the precursors of nucleic acids such as DNA and RNA and are implicated in the following biochemical and essential physiological processes: decoding genetic information, the mediation of energy metabolism, participation in cell signaling, and the modulation of long-chain polyunsaturated fatty acids.

In addition, β-glucan modifies immune responses (Ringø et al., 2012), with functions in the assimilation of essential nutrients for vitellogenesis, reproductive performance (Arshadi et al., 2018), lymphocyte activation and proliferation, phagocytosis in macrophages, immunoglobulin (Ig) response, intestinal microbiota regulation, cytokine expression (Gil, 2002), and disease resistance. Although nucleotides are not essential for fish as they are produced naturally, their presence is limited to tissues with rapid mitotic division (Rossi et al., 2007; Reda et al., 2018).

However, there are limited studies on the effects of the dietary co-supplementation of more than one immunomodulator, with no studies investigating the impacts of the dietary supplementation of high vs. “basal” levels of vitamins and the synergy of immunomodulators and vitamins. Therefore, in this study, we aimed to evaluate the hematoimmunological effects of the dietary co-supplementation of β-glucans+nucleotides and different levels of vitamins and the possible synergism between these factors on Nile tilapia after physical stress.

## Materials and methods

### Biological materials

Nile tilapia of the GIFT lineage, from a male mono-sex population, having an initial weight of 3.00 ± 0.68 g and a length of 5.33 ± 0.66 cm, were obtained from the Experimental Fish Farm of Camboriú, CEPC/EPAGRI-SC, Brazil. The immunomodulatory complex (Rovimax Boost) and the vitamin and mineral premix Optimum Vitamin Nutrition (OVN) were manufactured by DSM^®^, São Paulo, Brazil, and provided for the experiment. *Streptococcus agalactiae* S13 serotype Ib was isolated during an outbreak of mortality on a tilapia farm located in Paraná State by Facimoto et al. (2017) and was granted to the AQUOS Laboratory of the Federal University of Santa Catarina (UFSC). Its complete genome sequence is available in the DNA Data Bank of Japan/ European Molecular Biology Laboratory-Bank/ GenBank public databases under accession numbers CP018623 and PRJNA356737. Fish handling procedures were approved by the Ethics Committee on Animal Use of UFSC (CEUA/UFSC 2015231120).

### Experimental diets

In general, diets are formulated to meet the animals’ nutritional requirements; however, such formulations do not consider the effects of injuries that the animal may suffer during cultivation owing to bacterial, parasitic, and viral diseases and stress caused by inadequate handling and temperature fluctuations, thereby leading to casualties. In this study, we sought to provide experimental diets that meet the nutritional requirements of tilapia, as indicated in the Nutrient Requirements of Fish and Shrimp report published by the National Research Council (NRC), USA (Nutrient Requirements of Fish, 2011), and promote their resistance to stressful factors or adversities in the environment.

Four experimental diets were formulated to meet the nutritional requirements of the species, following the recommendations of Furuya (2010) and NRC (Nutrient Requirements of Fish ,2011) (Table 1). The diets produced were isocaloric and isoproteic with different levels of industrial premix and OVN (DSM^®^, Brazil) at 2.0 and 3.0 kg∙t^−1^, respectively, and the presence or not of the Rovimax Boost (DSM^®^, Brazil) immunostimulating complex at a concentration of 5.0 kg∙t^−1^ in the feed. The manufacturer’s recommendation for the inclusion of Premix Rovimax DSM^®^ was 1.5 to 2.0 kg∙t^−1^.

**Table 1:** Formulation and centesimal composition of experimental diets: feed with indicated level of vitamin (Vit _ind_); Vit _ind_ + Immunomodulator (Vit_indl+immune_); food with a high level of vitamin (Vit_high_); Vit_high_ + immunomodulator (Vit_high+immune_).

Ingredients (g kg^-1^)	Vit_ind_	Vit_indl+immune_	Vit_high_	Vit_high+immune_
Poultry by-product meal	155	155	155	155
Soybean meal	380	380	380	380
Corn	307.2	307.2	307.2	307.2
Corn gluten	55	55	55	55
Wheat bran	80	80	80	80
Dicalcium phosphate	7.4	7.4	7.4	7.4
DL-Methionine	4.6	4.6	4.6	4.6
Salt	2	2	2	2
Mineral premix1	30	30	30	30
A vitamin (KUI)	7500	7500	11000	11000
C vitamin (mg)	200	200	900	900
D vitamin (UI)	1500	1500	2000	2000
E vitamin (mg)	100	100	300	300
K vitamin (Menadiona) (mg)	3	3	10	10
Thiamine (mg)	2	2	20	20
Riboflavin (mg)	4	4	20	20
Niacin (mg)	35	35	120	120
Pantothenic acid (mg)	10	10	50	50
Pyridoxine (mg)	4	4	25	25
Biotin (mg)	0.1	0.1	1	1
Folic acid (mg)	4	4	7	7
Cyanocobalamin (mg)	0.02	0.02	0.05	0.05
Imunomodulator^2^	0^*^	50	0*	50
**Centesimal composition**^3^				
Dry matter (%)	90.42	89.98	90.43	90.49
Crude protein (%)	38	37.95	37.99	37.94
Digestible protein (%)	34.68	34.64	34.67	34.63
Ethereal extract (%)	5.2	5.17	5.19	5.17
Crude energy (kcal/kg)	4496.15	4476.09	4492.13	4472.08
Digestible energy (kcal/kg)	3701.28	3685.84	3698.19	3682.75
Crude fiber (%)	3.13	3.12	3.13	3.12
Ash (%)	6.08	6.08	6.08	6.08

Note. ^1^Mineral premix (0.3%): Copper (mg) 5.00; Iron (mg) 85.00; Manganese (mg) 25.00; Cobalt (mg) 0.05; Iodine (mg) 1.00; Zinc (mg) 80.00; Selenium (mg) 0.25;

2 Immunomodulator: Betaglucans (g.ton-1) 1000.00; Nucleotides (ppm) 150.00;

3 Proximate composition of dry matter.

* The empty spaces were filled with carbonate (CaCO_3_).

The diets were produced by extrusion in the form of 2 mm pellets. A horizontal mixer (Inbramaq, Riberão Preto, Brazil) was used to mix the dry ingredients, and extrusion was performed using a simple screw extruder MX40 (Inbramaq, Brazil). The conditions for extrusion were tested and adjusted in advance, and the extrusion parameters were as follows: the temperature in the cannon head was 85 °C, and a humidity level of 24% was attained with deionized water. After extrusion, the feed was oven-dried at 50 °C for 4.0 h and then packed and stored at −20 °C until use.

The determination of the centesimal composition of the diets was performed by the Nutrition Laboratory (LabNutri) of UFSC, following the standard protocol provided by the Association of Official Analytical Chemists _(_Baur & Ensminger, 1977). The dietary components measured included moisture levels (samples were dried at 105 °C until a constant weight was achieved, method 950.01), crude protein (Kjeldahl, method 945.01), ether extract (Soxhlet, method 920.39C), and mineral matter (incineration using muffle, method 942.05) (Table 1).

### Experimental design

A total of 560 Nile tilapia juveniles with an initial weight of 3.0 ± 0.68 g and length of 5.33 ± 0.66 cm, distributed in 28 polyethylene tanks having a usable volume of 80 L with 20 animals per tank and seven replicates for each treatment, were used. The fish were acclimatized for 15 days and fed the ration recommended by the producer during this period. After acclimatization, the animals were fed four different experimental diets for 60 days as follows:

- Feed with the indicated levels of vitamins (Vit_ind_),
- Feed with the indicated levels of vitamins +0.5% immunomodulator (Vit_ind_+Immune),
- Feed with high levels of vitamins (Vit_high_), and
- Feed with high levels of vitamins +0.5% immunomodulator (Vit_high_+Immune).

The fish were fed following the feeding table proposed by EPAGRI (Silva & Marchiori, 2018) based on the water temperature and size of the fish. Weekly biometric measurements were performed to monitor growth and adjust the feed supply. Excess food and excreta were removed from the tanks twice daily via siphoning.

During the entire experimental period, the tanks were coupled to a semi-open water recirculation system (recirculation aquaculture systems), equipped with mechanical and biological filtration, ultraviolet disinfection, and a 12 h photoperiod maintenance system (Owatari et al., 2018). The water quality parameters, such as hydrogenic potential (pH) and the levels of DO, total ammonia, toxic ammonia, and nitrite, were measured by the colorimetric method (Labcon^®^ test kit; Brazil), and the temperature was measured using a thermometer. All measured water quality parameters remained within the safe range for fish described by Leira et al. (2017) as follows: pH 7.3 ± 1.5, DO 6 ± 0.3 mg∙L^−1^; total ammonia 2.4 ± 1.3 mg∙L^−1^; toxic ammonia 0.006 ± 0.003 mg∙L^−1^; nitrite 0.30 ± 0.20 mg∙L^−1^, and temperature 26.5 ± 1.3 ºC.

After 60 days of supplementation, the fish weighed 30.40 ± 3.10 g, 34.25 ± 5.04 g, 34.12 ± 4.21 g, 34.12 ± 4.21 g, 36 ± 2.68 g in Vit_ind_, Vit_ind_+Immune, Vit_high_, and Vit_high_+Immune treatment groups, respectively, with a body length of 11.73 ± 4.23 cm, 12.02 ± 2.82 cm, 11.97 ± 5.13 cm, and 13.32 ± 3.44 cm in the respective groups. Subsequently, the fish were subjected to winter management, wherein the water temperature was adjusted to 20 °C (achieved within 12 h after adjustment), and two complete biometrics were performed. Later, the animals were subjected to physical stress wherein the maximum and minimum temperatures of the thermostat cycles in the tanks with the animals were set to 30 ºC and 20 ºC, resulting in three temperature fluctuations of 20 to 30 ºC, 30 to 20 ºC, and 20 to 30 ºC. All animals were exposed for 12 h to 30 ºC and 20 ºC temperatures each. At the end of the experimental period (post-stress), 140 fish were sampled for hematoimmunological analyses.

### Haematological analysis

At the end of the experiment, five fish per tank were anesthetized with eugenol Vetec^®^ (75 mg L^-1^), and the blood was withdrawn from the caudal vein using syringes with the anticoagulant ethylenediaminetetraacetic acid (EDTA 10%) for hematological analysis. Subsequently, blood smears were made in duplicate and stained with the May-Grunwald Giemsa Wright stain for differential leukocyte count and total thrombocyte count. Total leukocyte count (WBC) was obtained by the indirect method (Ranzani-Paiva et al., 2013) from blood smears. An aliquot of blood was used to determine the hematocrit (htc) (Goldenfarb et al., 1971), and another was used to quantify the total number of erythrocytes (RBC) in a Neubauer chamber after dilution 1:200 in Dacie solution modified according to Blaxhall & Daisley (1973). The analysis of hemoglobin (Hb), mean corpuscular hemoglobin (MCH), mean corpuscular hemoglobin concentration (MCHC), and mean corpuscular volume were realized using the equations by Ranzani-Paiva et al. (2013) and Glucose is obtained from blood plasma using a colorimetric test (Labteste®).

### Immunological analysis

The remaining blood used in the hematological analysis was allowed to coagulate for 1 h and centrifuged at 1400 *g* for 15 min at 4ºC to obtain blood plasma. The blood plasma was combined into pools of five fish per tank and stored at -20ºC for immunological analysis.

Total plasma protein (TPP) was measured using a commercial total protein kit (Lab Test^®^). Total immunoglobulin was measured according to the method of Amar et al. (2000), where 100 μL of plasma was added to 100 μL of 12% polyethylene glycol solution (PEG) (Sigma-Aldrich) and incubated at room temperature (24ºC) for 2 h for the precipitation of immunoglobulin molecules. The precipitate was removed by centrifugation (5000 *g* at 4ºC for 10 min). After the removal of the supernatant, the total protein amount was measured with a commercial kit (Lab Test^®^), and bovine albumin was used to construct a standard curve. The immunoglobulin concentration was expressed in mg mL^-1^ according to the following formula:


$$\text{Total immunoglobulin =}\left(\text{total protein in the serum - total protein PEG treated}\right)$$ (1)

Titration of agglutination activity was performed in 96-well U-bottom microplates by diluting the plasma in a 1:1 ratio in PBS in the first well (50 μL PBS solution:50 μL plasma) and performing serial 1:2 dilutions until the 12th well. Subsequently, 50 μL of inactivated *S. agalactiae* was added to all the wells. The microplate was incubated at 25°C for 18 h in a humidified chamber. Agglutination was confirmed by the observation of a precipitate in the bottom of the well and was considered as the reciprocal of the last dilution that presented agglutination (Silva et al., 2009).

The antimicrobial activity of plasma was determined against *S. agalactiae* in 96-well flat-bottom microplates according to Silva et al. (2009). The *S. agalactiae* inoculum was cultured in brain heart infusion (BHI) broth for 24 h at 28ºC, prepared at a concentration of 0.2 on the MacFarland scale and diluted in poor broth medium (PB) at 1×10^9^ UFC mL^-1^. The plasma was diluted in a 1:3 ratio in poor broth medium (PB) in the first well (50 μL plasma: 150 μL PB), and serial 1:2 dilutions were performed until the 12th well. For positive and negative controls, saline solution was diluted in PB, as was done with the plasma. Finally, 10 μL of *S. agalactiae* was added to the wells containing diluted plasma and the positive control. The microplates were incubated at 24 h at 28ºC. The microorganism growth was read in a microplate reader, at a wavelength of 550 ηm. The antimicrobial titer was the reciprocal of the last dilution that presented antibacterial activity with total inhibition of microbial growth.

### Statistical analysis

The data were subjected to Shapiro–Wilk and Levene tests to assess the normality and homoscedasticity of variance, respectively. Inhomogeneous data were transformed into log_10_ (×+1) values to achieve normality. Subsequently, data were subjected to a two-way analysis of variance for all analyses, and when appropriate, means were separated using Tukey’s test. All tests were performed at a 5% significance level using Statística version 10.0.

## Results

During the trials, including winter management and experimental stress, no mortality was observed, indicating strong stress resistance of the fish.

### Hematological analysis

The dietary addition of immunomodulators significantly increased the total leukocyte count and the number of lymphocytes (*P* <0.05). Regarding the effects of the different levels of vitamins, their high concentrations positively impacted the increase in hemoglobin (Hb) levels and mean corpuscular Hb (MCH) (*P* <0.05) (Table 2). However, the lowest Hb levels and MCH were observed in the group fed with the indicated vitamin levels.

**Table 2:** Hematological parameters of Nile tilapia (mean ± standard deviation) after 60 days with diets supplemented with: feed with indicated vitamin level (Vit_ind_); Vit_ind_ + Immunomodulator (Vit_ind+Immune_); food with a high level of vitamin (Vit_high_); Vit_high_ + Immunomodulator (Vit_high+Immune_) after a challenge with physical stress.

Parameters	Vit_ind_	Vit_ind+Immune_	Vit_high_	Vit_high+Immune_	*P*-value		
					Vitamin level	Immuno.	Inter.
Thr (×10^3^ µL^-1^)	4.59 ± 2.18	4.37 ± 2.50	5.08 ± 2.35	4.17 ± 2.24	0.716	0.175	0.407
WBC (×10^5^ µL^-1^)	2.34 ± 0.44^b^	2.53 ± 0.29^a^	2.33 ± 0.38^b^	2.37 ± 0.33^a^	0.141	0.046	0.199
Lym (×10^3^ µL^-1^)	218.64 ± 40.11^b^	236.26 ± 30.40^a^	218.01 ± 39.08^b^	225.40 ± 35.07^a^	0.311	0.030	0.366
Mon (×10^3^ µL^-1^)	11.61 ± 1.67	15.78 ± 1.69	11.47 ± 2.0	15.16 ± 1.52	0.874	0.111	0.918
Neu (×10^3^ µL^-1^)	2.17 ± 0.98	1.48 ± 0.45	1.97 ± 0.83	1.83 ± 1.04	0.922	0.489	0.614
Baso (×10^3^ µL^-1^)	1.10 ± 0.95	1.20 ± 0.98	1.10 ± 0.69	1.21 ± 1.00	0.544	0.205	0.505
RBC (×10^6^ µL^-1^)	2.34 ± 0.38	2.53 ± 0.29	2.33 ± 0.38	2.34 ± 0.33	0.095	0.079	0.139
Htc (%)	21.86 ± 5.02	24.34 ± 4.80	24.46 ± 4.73	23.81 ± 4.81	0.171	0.098	0.091
Hb (g dL^-1^)	6.7 ± 0.93^B^	6.86 ± 0.66^B^	7.72 ± 0.72^A^	7.19 ± 0.77^A^	0.029	0.540	0.256
Glucose (mg dL^-1^)	53.65 ± 6.98	49.52 ± 3.55	49.97 ± 4.94	52.69 ± 4.42	0.956	0.862	0.122
MCV (fL)	0.96 ± 0.21	0.97 ± 0.22	01.04 ± 0.17	01.01 ± 0.16	0.159	0.993	0.374
MCH (g dL^-1^)	0.29 ± 0.05^B^	0.27 ± 0.03^B^	0.33 ± 0.03^A^	0.31 ± 0.03^A^	0.004	0.066	0.756
MCHC (g dL^-1^)	31.09 ± 2.16	28.45 ± 3.02	31.03 ± 0.57	30.26 ± 1.56	0.193	0.102	0.616

Note. Different capital letters in the same line indicate statistical differences in the level of vitamin (A, B), and different lowercase letters in the same line indicate a statistical difference in the inclusion of immunomodulators (a, b). RBC: erythrocytes; Thr: thrombocytes; WBC: white blood cells; Lym: lymphocytes; Mon: monocytes; Neu: neutrophils; Baso: basophil; Htc: hematocrit; Hb: hemoglobin; MCV: mean corpuscular volume; MCH: mean corpuscular hemoglobin; MCHC: mean corpuscular hemoglobin concentration. Significance level 95%.

Thrombocyte, basophil, and neutrophil counts, as well as mean corpuscular volume, cell Hb concentration mean, hematocrit value, plasma glucose, and red blood cell counts were not affected (*P* >0.05) by any of the treatments in this study (Table 2).

### Immunological analysis

**Figure 1:**
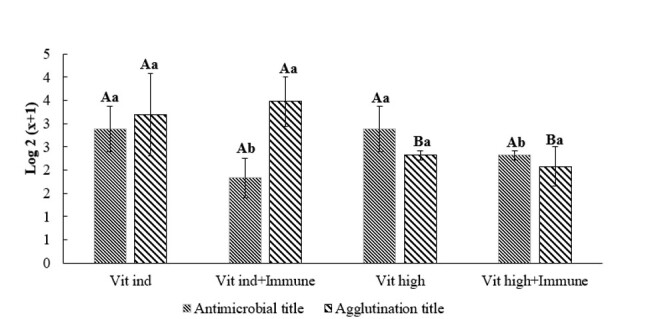
Antimicrobial title and agglutination tilapia of Nile tilapia (mean ± standard deviation) after 60 days with diets supplemented with: feed with indicated vitamin level (Vit_ind_); Vit _ind_ + immunomodulator (Vit_ind+Immune_); High vitamin level (Vit_high_); Vit_high_ + Immunomodulator (Vit_high+Immune_) after a challenge with physical stress

Note. Different capital letters indicate a statistical difference in the level of vitamin (A, B) and different lowercase letters indicate a statistical difference in the inclusion of immunomodulators (a, b).

After 60 days of supplementation and subsequent subjection to winter management and physical stress, the dietary addition of the immunomodulator promoted a significant increase in the levels of plasma Igs (*P* <0.05); however, it reduced the antimicrobial titer and total plasma protein (TPP) concentration (*P* <0.05). In contrast, the agglutination titer was impacted by the varying levels of vitamins (*P* <0.05). The antimicrobial titer was higher in the groups fed with diets without immunomodulators than in the groups fed with diets supplemented with immunomodulators (Figure 1). For the agglutination titer, a higher concentration was observed in the group that received the indicated levels of vitamins alone (3.33 ± 0.66 log_2_ [×+1]) than in the group that received high levels of vitamin alone (2.19 ± 0 .30 log_2_ [×+1]) (Figure 1).

**Figure 2:**
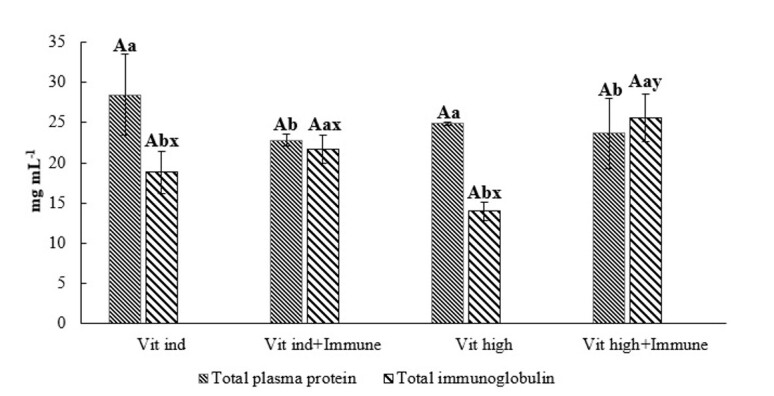
**.** Total plasma protein and immunoglobulin of Nile tilapia (mean ± standard deviation) after 60 days of diet supplemented with: feed with indicated level of vitamin (Vit_ind_); Vit_ind_ + immunomodulator (Vit_ind+Immune_); High vitamin level (Vit_high_); Vit_high_ + Immunomodulator (Vit_high+Immune_) after a challenge with physical stress.

Note**.** Different lowercase letters indicate statistical differences in the inclusion of immunomodulator (a, b) and letters (x, y) indicate an interaction between factors.

Immunoglobulin levels showed a significant difference both in response to the dietary inclusion of immunomodulators and also to the interactions of immunomodulators with the basal and high levels of vitamins (*P* <0.05) (Figure 2). When the immunomodulator inclusion factor was considered alone, the supplemented group was found to show higher mean levels of Ig (23.14 ± 2.88 mg∙mL^−1^) than that in the non-supplemented group (16.62 ± 3.22 mg∙mL^−1^). In the TPP levels, a significant difference (*P* <0.05) was observed upon the dietary inclusion of immunomodulators (Figure 2), with a higher concentration in the group fed with diets without the inclusion of immunomodulators (26.81 ± 4.01 mg∙mL^−1^) than in the group fed with diets supplemented with immunomodulators (23.18 ± 2.76 mg∙mL^−1^).

## Discussion

Fish fed with higher levels of vitamins had higher concentrations of Hb and MCH. The Hb molecule in fish and in other vertebrates can bind to and transport gases such as oxygen and carbon dioxide (di Prisco et al., 2007). The increase in the Hb levels and MCH may be associated with the levels of vitamin C incorporated into the diet, as treatments with higher levels of vitamins imply higher levels of vitamin C. Additionally, the stress to which the animals were subjected must have contributed to this effect. In addition, according to Mazur et al. (1960), vitamin C is involved in the release of iron stored in the form of ferritin in the liver and in the transport of plasma iron to the liver and its incorporation as ferritin in the tissue. Similar results were reported in studies by Lim et al. (2000) and Barros et al. (2002), wherein the effects of the interaction of vitamin C and iron on Hb concentration were observed in catfish (*Ictalurus punctatus*) and *O. niloticus*, respectively. Fernandes Junior et al. (2010) reported that Nile tilapia fed with different levels of choline (vitamin B8) also exhibit increased Hb levels.

There was a significant increase in the total leukocyte count and specifically in the number of lymphocytes in juvenile Nile tilapia fed with diets supplemented with immunomodulators. Immunomodulators such as β-glucans and nucleotides can increase the body’s immune response, providing resistance against bacterial, viral, and parasitic diseases and stress (Burrells et al., 2001; Miest et al., 2016).

The increase in the number of leukocytes and lymphocytes in fish fed with immunomodulator-supplemented diets may be associated with the ability of immunomodulators to modulate immune responses, which, in turn, increases the number of leukocytes, likely representing a better immune status. According to Moreira (2014), lymphocytes are immunocompetent cells that elicit immune responses. Similar results were reported by Sherif & Mahfouz (2019) in Nile tilapia fed with β-glucan, by Reda et al. (2018) studying the effects of nucleotides in Nile tilapia, and by Hassaan et al. (2018) in Nile tilapia fed with diets supplemented with yeast containing β-glucan and nucleotides.

Contrastingly, Barros et al. (2014) did not observe an increase in the number of lymphocytes and leukocytes in tilapia fed with β-glucan and vitamin C but observed a decrease in the number of these cells after transport stress. Sado et al. (2016) did not observe any differences in the number of these cells when an immunomodulator was included in the diet of Nile tilapia. The results reported by Barros et al. (2014) can be explained based on the time of dietary supplementation used in this study (7, 15, 30, and 45 days), whereas the results of Sado et al. (2016) may be associated with the supplementation route (bath and oral) and supplementation time (15 days). Both these methodologies differ from those used in this study.

It is also likely that this increase in the number of leukocytes is associated with both the immunomodulatory activity of dietary additives and stress response in animals, as mentioned by Miest et al. (2016), thus enhancing the immune response of animals fed with immunomodulator-supplemented diets.

According to Rauta et al. (2012), fish defense mechanisms are robust and are regulated by innate and specific immune systems. The innate system instantly provides the first line of defense wherein defense cells such as monocytes, lymphocytes, granular leukocytes, humoral components, and non-specific cytotoxic cells are involved. The specific system acts incisively and effectively and is linked to the capacity of lymphocytes to recognize antigens, but requires relatively more time to be activated. Antimicrobial titer, indicating the concentration of antimicrobial peptides, agglutination titer, and Ig and TPP levels in the blood serum are a few parameters indicative of the activity of the fish immune system. According to Uribe et al. (2011), the antimicrobial peptides act on the cell membranes of pathogens, inhibiting the activity of proteases, which, in turn, inhibits the action of bacterial toxins.

In this study, a significant increase was observed in the antimicrobial titer in fish that did not receive the dietary supplementation of immunomodulators. Similar results were reported by Ai et al. (2007) in a study with diets containing β-glucans for *Pseudosciaena crocea*, wherein the immunological parameters significantly decreased in animals treated with a high content of this peptide. These findings differ from those reported by Lin et al. (2011) and Amphan et al. (2019) in *Cyprinus carpio koi* and *O. niloticus*, respectively. They also differ from the findings of Koch et al. (2021), who observed an increase in the innate immune responses of *O. niloticus* fed with β-glucans; however, a reduction in these immune responses was observed during the 45 days of feeding.

Similarly, Misra et al. (2006) used 250 mg∙kg^−1^ of β-glucans in the diet of *Labeo rohita* and indicated an increase in immunological parameters until day 42 of the total experimental period (56 days). Bagni et al. (2005) observed no differences in the immunological parameters of *Dicentrarchus labrax* supplemented for 45 days with β-glucans, similar to the observations of Yamamoto et al. (2018) using β-glucans in the diet of *Sciaenops ocellatus*, Chagas et al. (2013) with *Colossoma macropomum*, and Berto et al. (2016) on the hematoimmunological responses of Nile tilapia to nucleotide enriched diets.

The time/duration factor and how the supplementation was administered may likely explain the results obtained in this trial, conducted for 60 days with oral supplementation. According to Sakai (1999), the effectiveness of immunomodulators decreases in the long term when using oral supplementation or immersion methods. Another likely explanation for these results is the enhanced immunological response in fish that did not receive the dietary supplementation of immunomodulators against the stress exerted on them.

Agglutination is a crucial parameter in immune and serum responses in fish, and the agents responsible for agglutination are lectins and Igs. Lectins are proteins capable of binding to sugars (through carbohydrate-binding sites) present in the cell membranes of pathogens that promote agglutination or opsonization (Vornholt et al., 2007; Magnadottir, 2010). However, Swain (2006) reported that the agglutination activity of Igs, produced by B lymphocytes, is greater than that of lectins, as Igs exhibit antigen-specific activity.

The agglutination titer was higher in treatments with the indicated dietary vitamin levels than in those with high levels of vitamins. However, Ig levels revealed an interaction between immunomodulators and the levels of vitamins in the diets and were found to be significantly increased with the dietary inclusion of immunomodulators. The findings of this study reveal that high vitamin content in the diets, in general, did not increase the agglutination titer of the animals. These results corroborate those of Peres et al. (2004), Lim et al. (2009), and Lim et al. (2010) in their research on Nile tilapia supplemented with vitamins E, inositol, C, and E, respectively. Despite the immunological importance of lectins found in the serum and mucus of fish, their relevance is not yet clear, but they may be linked to the fight against microorganisms (Nakamura et al., 2001; Tasumi et al., 2004).

The concentrations of Ig in the plasma of *O. niloticus* reveal an interaction between immunomodulators and the levels of vitamins present in the diets, with higher concentrations of Ig in the Vit_high_+Immune treatment group (quantitatively) than in the other groups. This result indicates a possible synergy between these factors, as the groups supplemented with high levels of vitamins or immunomodulators alone exhibited lower Ig concentrations than in the group co-supplemented with high levels of vitamins and immunomodulators. This finding may also be attributed to the high levels of vitamin C provided in the co-supplemented diet, as this vitamin has strong antioxidant properties, causing a protective effect against oxidative damage. Moreover, Dawood et al. (2017) and Wu et al. (2020) reported the synergistic effects of β-glucan with vitamin C and ascorbic acid in *Pagrus major* and *Epinephelus fuscoguttatus*, respectively.

In addition, the Ig levels in the groups fed with diets supplemented with immunomodulators were significantly higher than those in the groups fed with diets not supplemented with immunomodulators, showing a better response of this protein to the immunomodulators used in this study against the stress challenge. Analyzing the Ig concentrations in response to the basal or high dietary vitamin levels alone, we noticed that the concentrations decreased drastically in the Vit_ind_ and Vit_high_ treatment groups compared with that in the Vit_ind_+Immune and Vit_high_+Immune groups, respectively. Higher levels of Ig in the groups with the dietary inclusion of immunomodulators than in those without immunomodulators may be associated with an immune system response to the stresses suffered by these animals, as according to Vetvicka et al. (2013), β-glucans increase the immune response and stress resistance.

Lim et al. (2010) and Guimarães et al. (2014) also showed that vitamins E and A did not affect Ig production in *O. niloticus*. In the same study by Lim et al. (2010), higher levels of Ig were observed in fish fed with vitamin C-supplemented diets (200 mg∙kg^−1^) than in those fed with diets not supplemented with vitamin C; however, this increase was not significant when the fish were challenged with *S. iniae.*El-Murr et al. (2019) and Abdelhamid et al. (2020) observed that the dietary supplementation of β-glucans in *O. niloticus* increased the serum levels of M Ig (IgM) and globulins. Similarly, in *I. punctatus*, the serum levels of IgM increased upon the inclusion of 1 g∙kg^−1^ of β-glucans in their feed for 30 days (Phu et al., 2016).

Neamat‐Allah et al. (2020) reported a moderate increase in IgM levels in Nile tilapia when treated with β-glucans and stressed with atrazine (pesticide). Moreover, Reda et al. (2018), using nucleotides (0.25%) in the Nile tilapia diet, reported increased levels of IgM, but only in the initial 15 days, twice as long (30 days) when the immunomodulator had no significant effect. Murthy et al. (2009) reported that the co-feeding of β-glucans and nucleotides to shrimp resulted in better immune responses in these animals.

The TPP levels help determine the overall condition of fish. This parameter is highly sensitive to stress (Melo et al., 2009). The TPP levels are estimated by calculating the sum of the albumin and globulin contents in the plasma (Rodrigues et al., 2018). In this study, we observed that the TPP levels were affected by the dietary inclusion of immunomodulators; however, unlike that observed for Ig levels, the dietary inclusion of immunomodulators resulted in the lowest TPP levels. These results could be attributed to the better resistance of fish receiving the dietary supplementation of immunomodulators and the lowered resistance of those not receiving the supplementation of immunomodulators to stress exerted on them.

When analyzing the plasma glucose levels of the groups fed with diets without the inclusion of immunomodulators, an increase in plasma glucose concentration was observed, although not statistically significant, reinforcing the theory of increased stress suffered by the fish. These results are in agreement with those reported by Barros et al. (2015) for cold-stressed Nile tilapia, wherein the dietary inclusion of 0.8% β-glucans and 600 mg∙kg^−1^ vitamin C led to the lowest TPP and the highest globulin concentrations, characterizing a response to the stress.

Abdelhamid et al. (2020) confirmed that β-glucans tend to decrease the stress caused by diazinon (pesticide) in Nile tilapia. Melo et al. (2009) observed that male Nile tilapia subjected to hypoxia exhibited higher TPP concentrations than those in female Nile tilapia, and female Nile tilapia had higher concentrations of albumin and γ-globulin. In contrast, Almeida et al. (2018) reported that salt stress alone was insufficient to increase the TPP content of Nile tilapia, possibly owing to the age of the fish.

In recent studies with immunomodulators, Selim & Reda (2015) (3 g∙kg^−1^ of feed), Abu-Elala et al. (2018) (0.1% dietary inclusion), and El-Nobi et al. (2021) (symbiotic) reported an increase in TPP concentrations with the use of β-glucans in Nile tilapia culture. Moreover, Tahmasebi-Kohyani et al. (2012) reported increased TPP levels in rainbow trout (*Oncorhynchus mykiss*) with the inclusion of 0.15 and 0.2% of nucleotides in the diet and in response to acute stress (30 s out of water). In contrast, Souza et al. (2020) reported that the TPP levels in Nile tilapia were not affected by the inclusion of β-glucans in water, as well as that of the snapper (*P. major*) under oxidative stress caused by exposure to fresh water (Hossain et al., 2016), and in this study, the TPP levels decreased with the dietary inclusion of immunomodulators, possibly an immune stress response.

## Conclusion

We conclude that the inclusion of an immunomodulator in the diet of Nile tilapia provided resistance to physical stress experienced by these animals. In addition, it increased the number of leukocytes and lymphocytes in the treated animals. A possible synergism exists between immunomodulators and high levels of dietary vitamins, as indicated by the changes in Ig levels. It was also evident that high levels of vitamins in diets do not potentiate the immune response of fish against physical stress.

## References

[B001] Abdelhamid F. M., Elshopakey G. E., Aziza A. E. (2020). Ameliorative effects of dietary *Chlorella vulgaris* and β-glucan against diazinon-induced toxicity in Nile tilapia (*Oreochromis niloticus*). Fish & Shellfish Immunology.

[B002] Abdelrazek H. M. A., Tag H. M., Kilany O. E., Reddy P. G., Hassan A. M. (2017). Immuomodulatory effect of dietary turmeric supplementation on Nile tilapia (*Oreochromis niloticus*). Aquaculture Nutrition.

[B003] Abu-Elala N. M., Younis N. A., AbuBakr H. O., Ragaa N. M., Borges L. L., Bonato M. A. (2018). Efficacy of dietary yeast cell wall supplementation on the nutrition and immune response of Nile tilapia. Egyptian Journal of Aquatic Research.

[B004] Ai Q., Mai K., Zhang L., Tan B., Zhang W., Xu W., Li H. (2007). Effects of dietary β-1, 3 glucan on innate immune response of large yellow croaker, Pseudosciaena crocea. Fish & Shellfish Immunology.

[B005] Akanmu O. A., Enany S. (2018). Probiotics - Current Knowledge and Future Prospects.

[B006] Almeida D. M., Petesse M. L., Tachibana L., Dias D. C., Moreira R. G., Ranzani-Paiva M. J. T. (2018). Monitoring whole blood, plasma and serum variables of Nile tilapia during 24 hours, after capture stress. Boletim do Instituto de Pesca.

[B007] Altun T., Tekelioğlu N., Danabaş D. (2006). Tilapia culture and its problems in Turkey. E.U. Su Ürünleri Dergisi.

[B008] Amar E. C., Kiron V., Satoh S., Okamoto N., Watanabe T. (2000). Effects of dietary beta-carotene on the immune response of rainbow trout *Oncorhynchus mykiss.*. Fisheries Science.

[B009] Amphan S., Unajak S., Printrakoon C., Areechon N. (2019). Feeding-regimen of β-glucan to enhance innate immunity and disease resistance of Nile tilapia, *Oreochromis niloticus* Linn., against *Aeromonas hydrophila* and *Flavobacterium columnare.*. Fish & Shellfish Immunology.

[B010] Aramli M. S., Kamangar B., Nazari R. M. (2015). Effects of dietary β-glucan on the growth and innate immune response of juvenile Persian sturgeon, *Acipenser persicus.*. Fish & Shellfish Immunology.

[B011] Arshadi A., Yavari V., Oujifard A., Mousavi S. M., Gisbert E., Mozanzadeh M. T. (2018). Dietary nucleotide mixture effects on reproductive and performance, ovary fatty acid profile and biochemical parameters of female Pacific shrimp (*Litopenaeus vannamei*). Aquaculture Nutrition.

[B012] Associação Brasileira da Piscicultura (2024). Anuário 2024 Peixe BR da Piscicultura.

[B013] Bagni M., Romano N., Finoia M. G., Abelli L., Scapigliati G., Tiscar P. G., Sarti M., Marino G. (2005). Short- and long-term effects of a dietary yeast β-glucan (Macrogard) and alginic acid (Ergosan) preparation on immune response in sea bass (*Dicentrarchus labrax*). Fish & Shellfish Immunology.

[B014] Barros M. M., Falcon D. R., de Oliveira Orsi R., Pezzato L. E., Fernandes A. C., Guimarães I. G., Fernandes A., Padovani C. R., Sartori M. M. P. (2014). Non-specific immune parameters and physiological response of Nile tilapia fed β-glucan and vitamin C for different periods and submitted to stress and bacterial challenge. Fish & Shellfish Immunology.

[B015] Barros M. M., Falcon D. R., Orsi R. O., Pezzato L. E., Fernandes A. C., Fernandes A., de Carvalho P. L. P. F., Padovani C. R., Guimarães I. G., Sartori M. M. P. (2015). Immunomodulatory effects of dietary β-glucan and vitamin C in Nile tilapia, *Oreochromis niloticus* L., subjected to cold-induced stress or bacterial challenge. Journal of the World Aquaculture Society.

[B016] Barros M. M., Pezzato L. E., Kleemann G. K., Hisano H., Rosa G. J. M. (2002). Níveis de vitamina C e ferro para tilápia do Nilo (*Oreochromis niloticus*). Revista Brasileira de Zootecnia.

[B017] Barroso R. M., Tenório R. A., Pedroza M. X., Webber D. C., Belchior L. S., Tahim E. F., Carmo F. J., Muehlmann L. D. (2015). Gerenciamento genético da tilápia nos cultivos comerciais.

[B018] Baur F. J., Ensminger L. G. (1977). The Association of Official Analytical Chemists (AOAC). Journal of the American Oil Chemists’ Society.

[B019] Berto R. S., Pereira G. V., Mouriño J. L. P., Martins M. L., Fracalossi D. M. (2016). Yeast extract on growth, nutrient utilization and haemato-immunological responses of Nile tilapia. Aquaculture Research.

[B020] Blaxhall P. C., Daisley K. W. (1973). Routine haematological methods for use with fish blood. Journal of Fish Biology.

[B021] Bowyer P. H., El‐Haroun E. R., Hassaan M., Salim H., Davies S. J. (2019). Dietary nucleotides enhance growth performance, feed efficiency and intestinal functional topography in European Seabass (*Dicentrarchus labrax*). Aquaculture Research.

[B022] Brito J. M., Pontes T. C., Tsujii K. M., Araújo F. E., Richter B. L. (2017). Automação na tilapicultura: Revisão de literatura. Nutritime.

[B023] Burrells C., Williams P. D., Forno P. F. (2001). Dietary nucleotides: a novel supplement in fish feeds 1. Effects on resistance to disease in salmonids. Aquaculture (Amsterdam, Netherlands).

[B024] Chagas E. C., Pilarski F., Sakabe R., De Moraes F. R. (2013). Desempenho produtivo e respostas fisiopatológicas de tambaquis alimentados com ração suplementada com β-glucano. Pesquisa Agropecuária Brasileira.

[B025] Ching J. J., Shuib A. S., Abdul Majid N., Mohd Taufek N. (2020). Immunomodulatory activity of β‐glucans in fish: Relationship between β‐glucan administration parameters and immune response induced. Aquaculture Research.

[B026] Dawood M. A. O., Koshio S., Esteban M. Á. (2018). Beneficial roles of feed additives as immunostimulants in aquaculture: A review. Reviews in Aquaculture.

[B027] Dawood M. A. O., Koshio S., El-Sabagh M., Billah M. M., Zaineldin A. I., Zayed M. M., Omar A. A. E. D. (2017). Changes in the growth, humoral and mucosal immune responses following β-glucan and vitamin C administration in red sea bream, *Pagrus major.*. Aquaculture (Amsterdam, Netherlands).

[B028] Dawood M. A. O., Metwally A. E.-S., El-Sharawy M. E., Atta A. M., Elbialy Z. I., Abdel-Latif H. M. R., Paray B. A. (2020). The role of β-glucan in the growth, intestinal morphometry, and immune-related gene and heat shock protein expressions of Nile tilapia (*Oreochromis niloticus*) under different stocking densities. Aquaculture (Amsterdam, Netherlands).

[B029] di Prisco G., Eastman J. T., Giordano D., Parisi E., Verde C. (2007). Biogeography and adaptation of notothenioid fish: Hemoglobin function and globin–gene evolution. Gene.

[B030] Ding C., He J. (2010). Effect of antibiotics in the environment on microbial populations. Applied Microbiology and Biotechnology.

[B031] El-Murr A. E. I., Abd El Hakim Y., Neamat-Allah A. N. F., Baeshen M., Ali H. A. (2019). Immune-protective, antioxidant and relative genes expression impacts of β-glucan against fipronil toxicity in Nile tilapia, *Oreochromis niloticus.*. Fish & Shellfish Immunology.

[B032] El-Nobi G., Hassanin M., Khalil A. A., Mohammed A. Y., Amer S. A., Montaser M. M., El-sharnouby M. E. (2021). Synbiotic Effects of *Saccharomyces cerevisiae*, mannan oligosaccharides, and β-glucan on innate immunity, antioxidant status, and disease resistance of Nile tilapia, *Oreochromis niloticus.*. Antibiotics (Basel, Switzerland).

[B033] El‐Sayed A.-F. M., Izquierdo M. (2021). The importance of vitamin E for farmed fish - A review. Reviews in Aquaculture.

[B034] El-Sayed A.-F. M., Kawanna M. (2008). Optimum water temperature boosts the growth performance of Nile tilapia (*Oreochromis niloticus*) fry reared in a recycling system. Aquaculture Research.

[B035] El-Sayed A.-F. M. (2005). Tilapia culture.

[B036] Facimoto C. T., Chideroli R. T., Gonçalves D. D., do Carmo A. O., Kalaphotakis E., Pereira U. de P. (2017). Whole-Genome Sequence of *Streptococcus agalactiae* strain S13, isolated from a fish eye from a Nile tilapia farm in Southern Brazil. Genome Announcements.

[B037] Falcon D. R., Barros M. M., Pezzato L. E., Valle J. D. B. (2007). Lipid and vitamin C in practical diets preparatory for winter for Nile tilapia. Revista Brasileira de Zootecnia.

[B038] Fegan D. F., Lyons T. P., Jacques K. A., Hower J. M. (2006). Biotecnologia Nutricional nas Indústrias de Rações e Alimentos.

[B039] Fernandes A. C., Pezzato L. E., Guimarães I. G., Teixeira C. P., Koch J. F. A., Barros M. M. (2010). Resposta hemática de tilápias-do-nilo alimentadas com dietas suplementadas com colina e submetidas a estímulo por baixa temperatura. Revista Brasileira de Zootecnia.

[B040] Figueiredo H. C. P., Leal C. A. G. (2008). Tecnologias aplicadas em sanidade de peixes. Revista Brasileira de Zootecnia.

[B041] Furuya W. M. (2010). Tabelas Brasileiras para a Nutrição de Tilápias..

[B042] Gil A. (2002). Modulation of the immune response mediated by dietary nucleotides. European Journal of Clinical Nutrition.

[B043] Goldenfarb P. B., Bowyer F. P., Hall E., Brosious E. (1971). Reproducibility in the hematology laboratory: The microhematocrit determination. American Journal of Clinical Pathology.

[B044] Guimarães I. G., Lim C., Yildirim-Aksoy M., Li M. H., Klesius P. H. (2014). Effects of dietary levels of vitamin A on growth, hematology, immune response and resistance of Nile tilapia (*Oreochromis niloticus*) to *Streptococcus iniae.*. Animal Feed Science and Technology.

[B045] Hassaan M. S., Mahmoud S. A., Jarmolowicz S., El‐Haroun E. R., Mohammady E. Y., Davies S. J. (2018). Effects of dietary baker’s yeast extract on the growth, blood indices and histology of Nile tilapia (*Oreochromis niloticus* L.) fingerlings. Aquaculture Nutrition.

[B046] Hossain M., Koshio S., Ishikawa M., Yokoyama S., Sony N. M. (2016). Dietary nucleotide administration influences growth, immune responses and oxidative stress resistance of juvenile red sea bream (*Pagrus major*). Aquaculture (Amsterdam, Netherlands).

[B047] Koch J. F. A., de Oliveira C. A. F., Zanuzzo F. S. (2021). Dietary β-glucan (MacroGard^®^) improves innate immune responses and disease resistance in Nile tilapia regardless of the administration period. Fish & Shellfish Immunology.

[B048] Kraemer S. A., Ramachandran A., Perron G. G. (2019). Antibiotic pollution in the environment: From microbial ecology to public policy. Microorganisms.

[B049] Kubitza F. (2006). Atenção no manejo dos peixes na saída do inverno. Panorama da Aquicultura.

[B050] Leira M. H., Cunha L. T., Braz M. S., Melo C. C. V., Botelho H. A., Reghim L. S. (2017). Qualidade da água e seu uso em pisciculturas. Pubvet.

[B051] Li P., Zhao J., Gatlin 3rd D. M., Lee C.-S., Lim C., Gatlin 3rd D. M., Webster C. D. (2015). Dietary Nutrients, Additives, and Fish Health.

[B052] Lim C., Klesius P. H., Li M. H., Robison E. H. (2000). Interaction between dietary levels of iron andvitamin C on growth, hematology, immune response and resistance of channel catfish (*Ictalurus punctatus*) to *Edwardsiella ictaluri* challenge. Aquaculture (Amsterdam, Netherlands).

[B053] Lim C., Yildirim-Aksoy M., Li M. H., Welker T. L., Klesius P. H. (2009). Influence of dietary levels of lipid and vitamin E on growth and resistance of Nile tilapia to *Streptococcus iniae* challenge. Aquaculture (Amsterdam, Netherlands).

[B054] Lim C., Yildirim-Aksoy M., Welker T., Klesius P. H., Li M. H. (2010). Growth Performance, Immune Response, and Resistance to *Streptococcus iniae* of Nile Tilapia, *Oreochromis niloticus*, Fed Diets Containing Various Levels of Vitamins C and E. Journal of the World Aquaculture Society.

[B055] Lin S., Pan Y., Luo L., Luo L. (2011). Effects of dietary β-1,3-glucan, chitosan or raffinose on the growth, innate immunity and resistance of koi (*Cyprinus carpio* koi). Fish & Shellfish Immunology.

[B056] Lu D. L., Limbu S. M., Lv H. B., Ma Q., Chen L. Q., Zhang M. L., Du Z. Y. (2019). The comparisons in protective mechanisms and efficiencies among dietary α-lipoic acid, β-glucan and L-carnitine on Nile tilapia infected by *Aeromonas hydrophila.*. Fish & Shellfish Immunology.

[B057] Magnadottir B. (2010). Immunological control of fish diseases. Marine Biotechnology (New York, N.Y.).

[B058] Mazur A., Green S., Carleton A. (1960). Mechanism of plasma iron incorporation into hepatic ferritin. The Journal of Biological Chemistry.

[B059] McDowell L. R. (2000). Vitamins in animal and human nutrition.

[B060] Melo D. C., Oliveira D. A. A., Melo M. M., Júnior D. V., Teixeira E. A., Guimarães S. R. (2009). Perfil proteico de tilápia nilótica chitralada (*Oreochromis niloticus*), submetida ao estresse crônico por hipóxia. Arquivo Brasileiro de Medicina Veterinária e Zootecnia.

[B061] Miest J. J., Arndt C., Adamek M., Steinhagen D., Reusch T. B. H. (2016). Dietary β-glucan (MacroGard®) enhances survival of first feeding turbot (*Scophthalmus maximus*) larvae by altering immunity, metabolism and microbiota. Fish & Shellfish Immunology.

[B062] Misra C. K., Das B. K., Mukherjee S. C., Pattnaik P. (2006). Effect of long term administration of dietary β-glucan on immunity, growth and survival of *Labeo rohita* fingerlings. Aquaculture (Amsterdam, Netherlands).

[B063] Moreira C. (2014). Linfócitos. Revista de Ciência Elementar.

[B064] Murthy H. S., Li P., Lawrence A. L., Gatlin 3rd D. M. (2009). Dietary β-Glucan and nucleotide effects on growth, survival and immune responses of pacific white shrimp, *Litopenaeus vannamei.*. Journal of Applied Aquaculture.

[B065] Nakamura O., Watanabe T., Kamiya H., Muramoto K. (2001). Galectin containing cells in the skin and mucosal tissues in Japanese conger eel, Conger myriaster: An immunohistochemical study. Developmental and Comparative Immunology.

[B066] Neamat‐Allah A. N. F., El-Hakim Y. A., Mahmoud E. A. (2020). Alleviating effects of β‐glucan in *Oreochromis niloticus* on growth performance, immune reactions, antioxidant, transcriptomics disorders and resistance to *Aeromonas sobria* caused by atrazine. Aquaculture Research.

[B067] Nelson DL, Cox MM (2018). I principi di biochimica di Lehninger.

[B068] Nobrega R. O., Banze J. F., Batista R. O., Fracalossi D. M. (2020). Improving winter production of Nile tilapia: What can be done?. Aquaculture Reports.

[B069] Nutrient Requirements of Fish (2011). Nutrient requirements of fish and shrimp.

[B070] Owatari M. S., Jesus G. F. A., Brum A., Pereira S. A., Lehmann N. B., Pereira U. P., Martins M. L., Mouriño J. L. P. (2018). Sylimarin as hepatic protector and immunomodulator in Nile tilapia during *Streptococcus agalactiae* infection. Fish & Shellfish Immunology.

[B071] Penney J., Lu Y., Pan B., Feng Y., Walk C., Li J. (2019). Pure yeast beta-glucan and two types of yeast cell wall extracts enhance cell migration in porcine intestine model. Journal of Functional Foods.

[B072] Peres H., Lim C., Klesius P. H. (2004). Growth, chemical composition and resistance to *Streptococcus iniae* challenge of juvenile Nile tilapia (*Oreochromis niloticus*) fed graded levels of dietary inositol. Aquaculture (Amsterdam, Netherlands).

[B073] Phu T. M., Ha N. T. K., Tien D. T. M., Tuyen T. S., Huong D. T. T. (2016). Effect of beta-glucans on hematological, immunoglobulins and stress parameters of striped catfish (*Pangasianodon hypophthalmus*) fingerling. Can Tho University Journal of Science.

[B074] Pohlenz C., Gatlin 3rd D. M. (2014). Interrelationships between fish nutrition and health. Aquaculture (Amsterdam, Netherlands).

[B075] Prabu E., Rajagopalsamy C. B. T., Ahilan B., Jeevagan I. J. M. A., Renuhadevi M. (2019). Tilapia – an excellent candidate species for world aquaculture: A review. Annual Research & Review in Biology.

[B076] Ranzani-Paiva M. J., Pádua S. B., Tavares-Dias M., Egami M. I. (2013). Métodos para análise hematológica em peixes.

[B077] Rauta P. R., Nayak B., Das S. (2012). Immune system and immune responses in fish and their role in comparative immunity study: A model for higher organisms. Immunology Letters.

[B078] Reda R. M., Selim K. M., Mahmoud R., El-Araby I. E. (2018). Effect of dietary yeast nucleotide on antioxidant activity, non-specific immunity, intestinal cytokines, and disease resistance in Nile Tilapia. Fish & Shellfish Immunology.

[B079] Ringø E., Erik Olsen R., Gonzalez Vecino J. L., Wadsworth S. (2012). Use of immunostimulants and nucleotides in aquaculture: A review. Journal of Marine Science: Research & Development.

[B080] Ringø E., Zhou Z., Vecino J. L. G., Wadsworth S., Romero J., Krogdahl Å., Olsen R. E., Dimitroglou A., Foey A., Davies S., Owen M., Lauzon H. L., Martinsen L. L., De Schryver P., Bossier P., Sperstad S., Merrifield D. L. (2016). Effect of dietary components on the gut microbiota of aquatic animals. A never ending story?. Aquaculture Nutrition.

[B081] Rodrigues G. M., Nascimento F. G. de O., Bizare A., Oliveira W. J., Guimarães E. C., Mundim A. V. (2018). Serum biochemical profile of Nile tilapias (*Oreochromis niloticus*) bred in net cages during summer and winter. Acta Scientiae Veterinariae.

[B082] Rossi P., Xavier E., Rutz F. (2007). Nucleotídeos na nutrição animal. Revista Brasileira de Agrociência.

[B083] Sado R. Y., Gimbo R. Y., Salles F. B. (2016). Routes of β-glucan administration affect hematological and immune responses of *Oreochromis niloticus.*. Archivos de Zootecnia.

[B084] Sakai M. (1999). Current research status of fish immunostimulants. Aquaculture (Amsterdam, Netherlands).

[B085] Selim K. M., Reda R. M. (2015). Beta-glucans and mannan oligosaccharides enhance growth and immunity in Nile tilapia. North American Journal of Aquaculture.

[B086] Sherif A. H., Mahfouz M. E. (2019). Immune status of *Oreochromis niloticus* experimentally infected with *Aeromonas hydrophila* following feeding with 1, 3 β-glucan and levamisole immunostimulants. Aquaculture (Amsterdam, Netherlands).

[B087] Silva B. C., Marchiori N. (2018). Importância do manejo alimentar na criação de tilápia.

[B088] Silva B. C., Martins M. L., Jatobá A., Buglione C. C., Vieira F. N., Pereira G. V., Jerônimo P. G., Seiffert W. Q., Mouruño J. L. P. (2009). Hematological and immunological responses of Nile tilapia after polyvalent vaccine administration by different routes. Pesquisa Veterinária Brasileira.

[B089] Silva L. R., Rodhermel J. C. B., Andrade J. I. A., Pereira M. O., Chaaban A., Bertoldi F. C., Jatobá A. (2021). Antiparasitic effect of *Mentha* × villosa hydrolate against monogenean parasites of the Nile tilapia. Ciência Rural.

[B090] Siqueira R. P., Mello S. C. R. P., Jorge T. B. F., Seixas J. T., Pereira M. M. (2021). Viabilidade econômica da produção da tilápia do Nilo como atividade secundária em propriedades rurais no Estado do Rio de Janeiro. Research, Society and Development.

[B091] Souza F. P., de Lima E. C. S., Pandolfi V. C. F., Leite N. G., Furlan‐Murari P. J., Leal C. N. S., Mainardi R. M., Suphoronski S. A., Favero L. M., Koch J. F. A., Pereira U. P., Lopera-Barrero N. M. (2020). Effect of β-glucan in water on growth performance, blood status and intestinal microbiota in tilapia under hypoxia. Aquaculture Reports.

[B092] Swain P. (2006). P. Swain, P.K. Sahoo, S. Ayyappan. Fish and Shellfish Immunology: An Introduction, Narendra Publishing House, 1417, Kishan Dutt Street, Mali-Wara, Delhi-110006, India, 2006, ISBN 81-85375-90-9. Fish & Shellfish Immunology.

[B093] Tahmasebi-Kohyani A., Keyvanshokooh S., Nematollahi A., Mahmoudi N., Pasha-Zanoosi H. (2012). Effects of dietary nucleotides supplementation on rainbow trout (*Oncorhynchus mykiss*) performance and acute stress response. Fish Physiology and Biochemistry.

[B094] Tasumi S., Yang W.-J., Usami T., Tsutsui S., Ohira T., Kawazoe I., Wilder M. N., Aida K., Suzuki Y. (2004). Characteristics and primary structure of a galectin in the skin mucus of the Japanese eel, *Anguilla japonica.*. Developmental and Comparative Immunology.

[B095] Uribe C., Folch H., Enriquez R., Moran G. (2011). Innate and adaptive immunity in teleost fish: A review. Veterinarni Medicina.

[B096] Vetvicka V., Vannucci L., Sima P. (2013). The effects of β - glucan on fish immunity. North American Journal of Medical Sciences.

[B097] Vornholt W., Hartmann M., Keusgen M. (2007). SPR studies of carbohydrate–lectin interactions as useful tool for screening on lectin sources. Biosensors & Bioelectronics.

[B098] Wang A., Ran C., Wang Y., Zhang Z., Ding Q., Yang Y., Olsen R. E., Ringø E., Bindelle J., Zhou Z. (2019). Use of probiotics in aquaculture of China: A review of the past decade. Fish & Shellfish Immunology.

[B099] Watts J. E. M., Schreier H. J., Lanska L., Hale M. S. (2017). The rising tide of antimicrobial resistance in aquaculture: sources, sinks and solutions. Marine Drugs.

[B100] Wismar R., Brix S., Frøkiaer H., Laerke H. N. (2010). Dietary fibers as immunoregulatory compounds in health and disease. Annals of the New York Academy of Sciences.

[B101] Wu B., Wang Q., Cao J., Mei J., Xie J. (2020). Effects of ascorbic acid and β-1,3-glucan on survival, physiological response and flesh quality of cultured tiger grouper *(Epinephelus fuscoguttatus*) during simulated transport in water. Biology (Basel).

[B102] Xu L., Ran C., He S., Zhang J., Hu J., Yang Y., Du Z., Yang Y., Zhou Z. (2015). Effects of dietary yeast nucleotides on growth, non-specific immunity, intestine growth and intestinal microbiota of juvenile hybrid tilapia *Oreochromis niloticus* × *Oreochromis aureus.*. Animal Nutrition.

[B103] Yamamoto F. Y., Yin F., Rossi W., Hume M., Gatlin 3rd D. M. (2018). β-1,3 glucan derived from Euglena gracilis and Algamune^TM^ enhances innate immune responses of red drum (*Sciaenops ocellatus* L.). Fish & Shellfish Immunology.

[B104] Zadinelo I. V., Carneiro W. F., Balen R. E., Oenning J. P., Meurer F. (2020). Avaliação de rações comerciais para a tilápia do Nilo durante o período de outono/inverno. Nutritime.

[B105] Zanolo B., Yamamura H. (2006). Parasitas em tilápias-do-nilo criadas em sistema de tanques-rede. Ciências Agrárias.

[B106] Zerai D. B., Fitzsimmons K. M., Collier R. J. (2010). Transcriptional response of delta-9-desaturase gene to acute and chronic cold stress in Nile tilapia, *Oreochromis niloticus.*. Journal of the World Aquaculture Society.

